# The Importance of Th2 Immune Responses in Mediating the Progression of Gastritis-Associated Metaplasia to Gastric Cancer

**DOI:** 10.3390/cancers16030522

**Published:** 2024-01-25

**Authors:** Giuseppe Privitera, Joseph J. Williams, Carlo De Salvo

**Affiliations:** 1 Department of Pathology, Case Western Reserve University School of Medicine, Cleveland, OH 44106, USA; gpp.privitera@icloud.com (G.P.); jxw1519@case.edu (J.J.W.); 2 Dipartimento di Scienze della Salute, Università degli Studi di Milano, 20142 Milan, Italy

**Keywords:** gastric metaplasia, spasmolytic polypeptide-expressing metaplasia (SPEM), intestinal metaplasia, IL-33, Th2 immunity, M2 macrophages, eosinophils

## Abstract

**Simple Summary:**

Gastric cancer is one of the most prevalent and deadliest neoplasms worldwide. Although significant advances have been made in recent decades to improve its treatment, an incomplete understanding of its etiology and pathogenesis still limits our ability to effectively prevent and treat gastric cancer. It is well established that the most common subtype of gastric cancer (intestinal gastric cancer) develops through a multi-step process, wherein pathologic changes in gastric cells progressively occur, ultimately leading to the development of tumors. Recent evidence suggests that, aside from environmental factors, the immune system plays a key role in the sequela from gastritis to metaplasia, dysplasia and finally, gastric cancer, primarily through type 2 immune responses. In this review, we summarize the current literature and provide an overall interpretation regarding the impact of T helper 2 (Th2) immunity on the development and progression of gastric cancer.

**Abstract:**

Gastric cancer is one of the leading causes of cancer deaths worldwide, with chronic gastritis representing the main predisposing factor initiating the cascade of events leading to metaplasia and eventually progressing to cancer. A widely accepted classification distinguishes between autoimmune and environmental atrophic gastritis, mediated, respectively, by T cells promoting the destruction of the oxyntic mucosa, and chronic *H. pylori* infection, which has also been identified as the major risk factor for gastric cancer. The original dogma posits Th1 immunity as a main causal factor for developing gastritis and metaplasia. Recently, however, it has become evident that Th2 immune responses play a major role in the events causing chronic inflammation leading to tumorigenesis, and in this context, many different cell types and cytokines are involved. In particular, the activity of cytokines, such as IL-33 and IL-13, and cell types, such as mast cells, M2 macrophages and eosinophils, are intertwined in the process, promoting chronic gastritis-dependent and more diffuse metaplasia. Herein, we provide an overview of the critical events driving the pathology of this disease, focusing on the most recent findings regarding the importance of Th2 immunity in gastritis and gastric metaplasia.

## 1. Introduction

In 2020, gastric cancer was ranked 6th among cancers in regard to age-standardized incidence rate and was the 5th leading cause of cancer deaths worldwide [1]. Gastric adenocarcinoma represents the most common form, with two main histological variants described: ‘intestinal’ and ‘diffuse’ gastric cancer, with the former being the most prevalent and associated with lower mortality [2]. Diffuse gastric cancer is characterized by loss of cell–cell adhesions secondary to alterations in the adhesion molecule, E-cadherin, which leads to the absence of organized glandular structures [3]. While no precursor lesion has been identified for the diffuse type, intestinal gastric cancer is thought to arise through a well-established sequence of events that consists of inflammation–atrophy–metaplasia-dysplasia–invasive carcinoma. This sequence, also known as the ‘Correa cascade’ (i.e., acute gastritis–chronic gastritis–metaplasia–dysplasia–cancer), has been best characterized in chronic gastritis secondary to *H. pylori* infection [4] but can be similarly applied to other chronic gastric conditions that cause mucosal atrophy.

Neutrophils are considered to be the main effector cells of the acute/innate immune responses against *H. pylori* [5,6,7], while it was originally believed that the main chronic/adaptive immune responses driving *H.pylori*-dependent gastritis and gastric metaplasia were of the T helper (Th) 1 type, involving systemic and mucosal *H. pylori*-specific antibody production and Th1-type cytokine production [8,9,10,11,12,13,14,15]. In the event that these effector cells fail to eradicate *H. pylori*, chronic gastric inflammation and mucosal damage are thought to ensue. Additionally, antibodies can contribute to epithelial damage by activating the complement cascade and through immune complex-mediated mechanisms [16,17], whereas T cell responses can induce or perpetuate tissue and epithelial damage, as also suggested by the decreased incidence of *H. pylori* gastritis in patients with AIDS [17,18,19].

*Autoimmune vs. Atrophic Gastritis as Precursors of Gastric Metaplasia*

Atrophy of the gastric mucosa emerges as a long-term consequence of uncontrolled gastric inflammation and is characterized by the loss of specialized gastric cells [4]. A widely accepted classification distinguishes between autoimmune and environmental atrophic gastritis. In autoimmune gastritis (AIG), T cells mediate the destruction of the oxyntic mucosa (i.e., mucosa localized to the fundus and body of the stomach, with glands mainly containing parietal and chief cells), likely by targeting hydrogen–potassium ATPase expressed by parietal cells [20], with prominent eosinophilic infiltration observed [21]. Severe destruction of the oxyntic mucosa is associated with the development of pernicious anemia (megaloblastic anemia secondary to vitamin B12 deficiency) and an increased risk of gastric adenocarcinoma and neuroendocrine tumors [22].

Pathogenic Th2 responses in AIG have been implicated by previous studies illustrating activated Th1 and Th2 autoantigen-specific T cells in the regional lymph nodes of day 3 thymectomized (d3tx) BALB/c mice, using an autoimmune disease-prone model, wherein both H^+^K^+^ATPase-specific Th1 and Th2 cell clones are able to transfer severe disease; a TCR transgenic mice generated from these T cell clones spontaneously develops AIG [23]. The pathogenicity of the Th2 response is supported by a significant reduction in disease and immune responses in *Il4^−/−^* and eosinophil-deficient mice, in which the pathology is mainly attributed to reduced parietal cell loss and mucin cell hyperplasia and correlates with reduced eosinophil infiltration in the inflamed stomach. Of note, it would appear that loss of T regulatory cell (Treg) function is crucial to promote Th2 responses in this context, as AIG presents with dominant Th2 features in DEREG (Depletion of REGulatory T cells) mice with transient Treg depletion; interestingly, it is speculated that dendritic cells play a key role in eliciting Th2 immune responses in this model [24]. On the other hand, prolonged and elevated levels of IL-33 can induce proliferation and infiltration of eosinophils into the gastric mucosa, which are key players in the development of intestinal-like metaplasia and promote an underlying chronic inflammatory state in the stomach that can create favorable conditions for the transition from intestinal metaplasia to gastric cancer [25]. Eosinophils may also promote the induction of Th2-dependent antibody responses in the lymph nodes and directly present antigens to T cells while releasing cytokines that promote Th2 differentiation [26,27]. Moreover, the depletion of Tregs can promote the expansion of eosinophils within draining lymph nodes during autoimmune gastritis, leading to a dominant Th2 response [24], suggesting a higher susceptibility to Th2 cell apoptosis in the presence of Tregs and, therefore, tighter Treg control of Th2 cells compared to Th1 cells [28]. In fact, a shift from Th1- to Th2-mediated immune responses is also found in patients with gastric cancer and dysplasia [29].

The most common risk factor for environmental atrophic gastritis is chronic *H. pylori* infection, which is also identified as the major risk factor for gastric cancer [30]. Of note, dietary factors (namely, diets rich in salts) have also been reported to contribute to the development of chronic atrophic gastritis [31]. Early *H. pylori* infection elicits an innate immune response that is primarily—but not solely—mediated by IL-8 [32], which is produced by epithelial cells and acts as a chemotactic factor and activator for neutrophils; notably, such a response is primarily dependent on intracellular receptors of gastric epithelial cells, as *H. pylori* antigens evade human TLRs 4 and 5, which are normally activated by flagellins and lipopolysaccharides of other Gram-negative bacteria [33]. As the inflammation progresses, monocytes are recruited and activated towards an M1 phenotype (typical of Th1 responses) to produce IL-1β, IL-6 and IL-8 [34]; furthermore, M1 macrophages also produce IL-12, which contributes to the activation of Th1 cells [35] that establish a chronic inflammatory environment, characterized by increased levels of several proinflammatory cytokines, among which IL-17 is one of the most represented [36]. In chronic *H. pylori* chronic infection, atrophy is typically multifocal and commonly localized to the antrum [4]. Timely eradication of *H. pylori* during the early phases of gastritis and before chronic changes of the gastric mucosa significantly reduces the risk of developing gastric cancer. A more delayed eradication can, at least partially, revert mucosal atrophy and reduce the risk of malignant transformation; however, once severe mucosal transformation has been established, eradication is not beneficial for reducing the risk of cancer development [37]. In general, a balanced Th1/Th2 immune response is found in the majority of cases with non-atrophic chronic gastritis, while a prevalent Th2 immune response, mediated by IL-13 secreting cells, is observed in patients with intestinal metaplasia and intestinal-type gastric cancer [38], indicating that a shift from Th1 to Th2 immune response, and in particular IL-13 secretion, may be involved in the different outcomes of *H. pylori* infection and its progression towards carcinogenesis. Indeed, it has been shown that differential polarization in immune responses towards Th1 vs. Th2 influences the outcome of *H. pylori* infection; specifically, while Th1 immune responses are seemingly associated with more florid inflammation and increased bacterial clearance, Th2 immune responses, on the other hand, appear to induce less robust inflammation, with reduced bacterial clearance and an increased risk of cancer development [39].

Considering the role that Th2 immunity and eosinophils play in allergy and in response to parasitic infection, it seems reasonable to presume that *H. pylori* infection, at some point, may also elicit this type of immune response. Overall, the complexity of crosstalk between different immune responses during gastritis and gastric metaplasia is quite evident, and the prominent role of Th2 immune responses and the cell types involved in Th2 immunity have emerged in recent years. Chronic gastritis is associated with an increased risk for the development of gastric cancer [40], and studies have shown that non-resolving inflammation is mechanistically linked to neoplastic transformation in the stomach. It is important to note that the specific cellular and molecular mechanisms of acute-to-chronic gastritis and gastric cancer are important aspects of disease progression to study; however, these pathways have been extensively reviewed elsewhere [41] and are beyond the scope of the present review. Instead, we will focus our review on the early events associated with gastritis and gastric metaplasia.

## 2. Inflammation in the Stomach and Gastric Metaplasia

### 2.1. Gastric Metaplasia Occurs in Different Forms, Possibly Representing Separate Stages of Carcinogenesis

Metaplasia often accompanies atrophy in chronic gastritis, which are both considered pre-cancerous conditions [42]. Metaplasia is defined as the substitution of terminally differentiated cells with other terminally differentiated cells that are normally not present in a specific anatomic location. Two main forms of metaplasia of the gastric mucosa are described: intestinal metaplasia [30] and pseudopyloric metaplasia (also referred to as spasmolytic polypeptide-expressing metaplasia or SPEM) [43]. In intestinal metaplasia, the oxyntic and/or antral mucosa is replaced by the intestinal epithelium, notably with the abundant representation of intestinal goblet cells. Intestinal metaplasia can be further classified as follows: (1) complete (resembling small intestinal epithelium), characterized by the presence of enterocytes with a brush border, Paneth cells and goblet cells that produce intestinal sialomucins (primarily only MUC2, while the typical gastric mucins, MUC5 and MUC6, are absent [44,45]), or (2) incomplete (resembling colonic mucosa), with a co-mixture of intestinal goblet cells (producing either sialomucins or sulfomucins) and gastric goblet cells (producing neutral mucins), but with neither enterocytes nor Paneth cells. Importantly, incomplete metaplasia is reported to represent a more advanced stage of metaplasia that is associated with a higher risk of progression to gastric cancer [46]. The other form of metaplasia, SPEM, is characterized by a high expression of trefoil factor family 2 (TTF2, previously known as spasmolytic peptide) and by the substitution of parietal and chief cells in the oxyntic mucosa with mucus-secreting epithelial cells that are normally present in the antrum of the stomach [43]. It is reported that SPEM can precede intestinal metaplasia [47].

The interest in SPEM has grown throughout the years, and in fact, quite recently, SPEM has been characterized in different mouse models of gastric cancer [48,49,50,51,52]. Notably, the concept that SPEM is a neoplastic precursor of the downstream carcinogenesis cascade is supported by evidence in patients and murine models, whereby intestinal metaplasia develops in the presence of pre-existing SPEM [53,54,55].

Nonetheless, controversy still exists around the exact sequence of events that characterize the development of SPEM. Currently, the most well-accepted hypothesis is that SPEM arises from transdifferentiation of chief cells that concomitantly occurs with parietal cell loss [56]. However, it is also thought that neck and progenitor cells at the gland isthmus might be responsible for the development of SPEM, at least under specific conditions [57]. A recent study performed via trajectory analysis of primary gastric adenocarcinoma cells suggests that gastric neoplastic cells may originate from transdifferentiated chief cells [58], further reinforcing the notion that chief cells are key in the pathogenesis of intestinal gastric cancer. In fact, transdifferentiation of chief cells to SPEM cells has been described as a multi-step process. First, normal cell morphology is disrupted through the activation of lysosome and autophagy pathways [59]. Then, TFF2, which can bind MUC6 in mucin-containing granules, is expressed [60], and CD44v9 is upregulated [61]. In parallel, a number of transcription factors are up- and down-regulated [62] as cellular genomic and transcriptomic rearrangement occurs. Finally, a minority of SPEM cells may express mTORC1 to acquire a proliferative phenotype, while most SPEM cells are usually non-proliferative [59]. Importantly, it has been reported that in Mist1-Kras mice, metaplastic transition is dependent on the Ras signaling pathway; in fact, Kras activation in chief cells induces SPEM and downstream intestinal metaplasia, and treatment with a MEK inhibitor leads to regression of intestinal metaplasia and restoration of normal mucosal homeostasis [63]. In addition, aquaporin-5 (AQP5) has recently been identified as a novel marker of SPEM and, interestingly, its expression is also observed at the base of the glands in incomplete intestinal metaplasia, but it is not detected in the complete form [64].

### 2.2. Different Murine Models Help to Understand Different Aspects of Metaplasia Progression to Overt Cancer

Understanding which signals control and which processes regulate SPEM transdifferentiation is crucial in terms of dissecting the pathogenic pathways of intestinal gastric cancer. In this regard, different models can aid in the investigation of potential mechanism(s) from different points of view and stages of disease. Drug-induced models of gastritis have facilitated important discoveries regarding the role of the immune system in the development of SPEM, as well as the progression to more advanced forms of epithelial lesions (i.e., intestinal metaplasia, dysplasia and cancer). Studies with diphtheria toxin reveal that parietal cell destruction is not sufficient to induce SPEM [65], thereby suggesting that immune signals of some sort are also necessary for cell transdifferentiation. The drugs DMP-777 and L635 cause acute parietal cell death due to their actions as parietal cell-targeted protonophores [66]; similar toxic effects are obtained using high-dose tamoxifen [67]. Acute recruitment of M2 macrophages and the upregulation of proinflammatory type 2 cytokines occur after drug-induced acute parietal cell loss, which represent hallmark features of SPEM [68,69]. Importantly, out of the three, only L635 induces substantial inflammation, which is likely required for SPEM to progress toward carcinogenesis [70].

More sophisticated models provide support for this concept. For instance, some of the first models of *Helicobacter (H.)*-associated gastric metaplasia used long-term infection with either *H. felis* or *H. pylori* strains, such as pre-mouse Sydney Strain 1 (PMSS1), which are able to trigger chronic inflammation, atrophy, and mucus-producing gastric metaplasia [71,72,73,74,75,76]. In T cell-deficient mice, *H. felis* does not induce any evident gastric pathology despite high levels of colonization, whereas in Myd88-deficient mice with impaired immunological tolerance, gastric pathology is accelerated [73,77,78], suggesting that, in these models of *Helicobacter* infection, atrophy and metaplasia are due to the development of host immune and inflammatory responses. On the other hand, obesity-induced, low-grade systemic inflammation exacerbates *H. Felis*-dependent gastric pathology by enhancing type 17 T-helper cell (Th17) responses [79]. However, germ-free mice develop attenuated inflammation and metaplasia [80], suggesting that the commensal gastric microflora may play a role in disease progression.

Similar to DMP-777-induced SPEM, KLF4-deficient mice develop SPEM in the absence of inflammation that does not progress to intestinal metaplasia, leading to dysplasia [49]. On the other hand, mice lacking *Hip1r* in parietal cells develop parietal and chief cell loss and a SPEM lineage accompanied by inflammatory infiltrates at 5 weeks of age [48,81]; these models, however, do not express markers of intestinal metaplasia, such as Muc2 and villin. The K19-C2mE transgenic mice and its Wnt1-expressing (K19-Wnt1/C2mE or Gan mice) variant develop SPEM at 12 and 5 weeks of age and progress to tumors at 48 and 20 weeks, respectively [50,51]; these mice present with mucosal lesions associated with prominent macrophage infiltrates similar to *H. felis*-induced SPEM. Quite interestingly, mice deficient for *Runx3* develop a SPEM phenotype, without inflammation and without loss of parietal cells; however, cells at the base of some SPEM glands express the intestinal markers, Muc2 and CDX2, that are normally absent in other gastric metaplasia models [52]. Therefore, the comparison of different models provides support and reference points that can be used and interpreted in the context of other models. More recently, the senescence-accelerated prone mouse 1, or SAMP1/YitFc (SAMP) mouse strain, was employed to study the development of SPEM and progression to proliferative intestinal metaplasia [25]. SAMP mice represent a model of spontaneous Crohn’s disease-like ileitis, which also displays a progressive, chronic immune-mediated gastritis that is preceded by an abnormally high, region-specific increase in gastric epithelial permeability. SAMP gastritis occurs independently of *Helicobacter* infection and in fact, uniquely persists in the absence of commensal flora [82]. Chronic gastritis-prone SAMP mice progressively develop metaplasia, leading to advanced SPEM-like features [25,83].

### 2.3. Chronic Inflammation and Metaplasia Are Key Processes for Advancement to Gastric Carcinogenesis

Inflammation, in fact, is a general immune-mediated response in tissues reacting to infection, injury or any insult that affects homeostasis and results in traumatic changes in tissue structure and functionality. Similarly, metaplasia has been proposed to evolve as a defense mechanism, allowing the mucosal surface to assume a protective, mucus-producing phenotype in the presence of insulting factors [84]. Therefore, it is not surprising that inflammation and metaplasia can occur simultaneously within the injured stomach. Concomitantly with such processes, the immune system can also initiate anti-inflammatory/protective responses to counteract and resolve the ongoing inflammation, which include wound healing and active tissue repair processes [85]. Therefore, investigating the mechanism(s) of inflammation and the different steps leading to gastric cancer may provide a powerful tool for understanding cancer development and prognosis.

**Figure 1:**
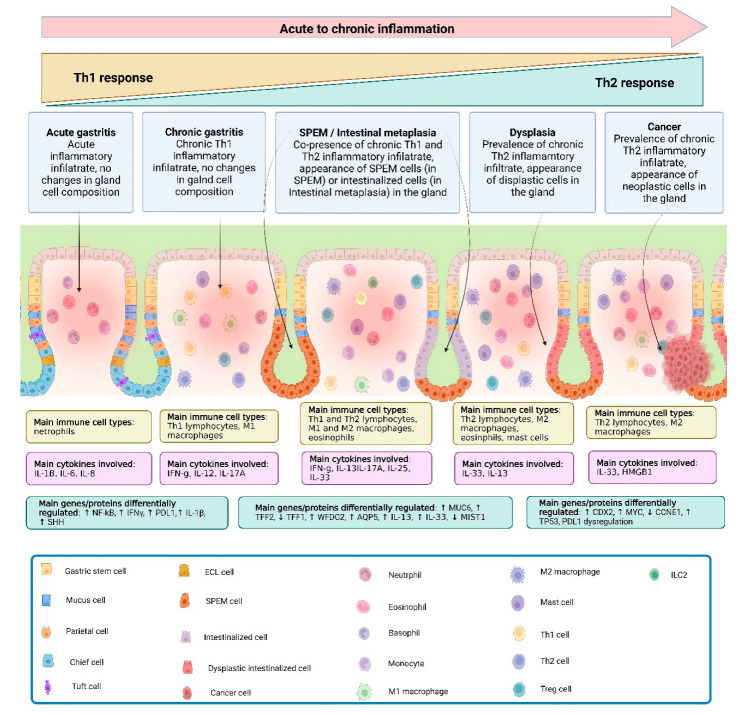
**Progression from gastritis to gastric cancer.** At the onset of gastric inflammation and during its progression to a chronic stage, neutrophils and Th1-associated mediators and cell types are prevalent. As chronic inflammation proceeds, multiple cell types infiltrate the gastric mucosa as part of a mixed Th1/Th2 immune response, leading to parietal cell loss and progressive chief cell transdifferentiation, with an increase in the spasmolytic peptide, TFF2, resulting in SPEM. If the chronic inflammatory insult does not subside, SPEM progresses to a more intestinalized phenotype, with the proliferation of intestinal mucus-secreting goblet-like cells, a full Th2 response and the initiation of an irreversible stage of disease, which can eventually lead to dysplasia and cancer. Abbreviations: CCNE1, Cyclin E1; CDX2, homeobox protein CDX-2; IFN, interferon; IL, interleukin; AQP5, aquaporin 5; MIST1, muscle, intestine and stomach expression 1; MUC6, mucin 6; MYC, Myc proto-oncogene protein; NK-kB, nuclear factor kappa-light-chain-enhancer of activated B cells; PD-L1, programmed death-ligand 1; SHH, sonic hedgehog; TFF2, trefoil factor 2; TP53, tumor protein P53; WFDC2, WAP four-disulfide core domain 2.

Under the condition of persistent, non-resolving chronic inflammation, tissue repair processes can become dysregulated and lead to metaplastic events that can eventually progress to cancer. Chronic inflammation, in fact, represents the first step in both intestinal and diffuse types of gastric cancer and likely plays a pivotal role in the pathogenic progression to gastric cancer. In this context, macrophages are involved in generating a chronic inflammatory microenvironment and are persistently activated, leading to continuous tissue damage [86]. In the context of chronic inflammation, a metaplastic mucosa can acquire a proliferative phenotype that can potentially lead to dysplasia and cancer. [87], with increasing evidence suggesting that chief cells undergo reprogramming following injury that leads to gastric tissue repair [56,57,66,88]. When this repair process is combined with chronic injury and inflammation, it can lead to metaplasia and dysplasia (Figure 1).

SPEM/intestinalized SPEM is considered to be a precursor of dysplastic events and downstream carcinoma, and it may not be reversible in the presence of chronic inflammation [4]. Non-resolving chronic inflammation, often related to *H. pylori* infection, is pivotal in the progression from gastric epithelial transdifferentiation to intestinal-like gastric metaplasia [4,89].

## 3. Th2 Pathways in Gastric Metaplasia

### 3.1. Th2 Immune Responses, Specifically via IL-13, Is Central to the Pathogenesis of Gastric Cancer

In *H. pylori*-related inflammatory responses, a predominant Th1 response is present in the majority of *H. pylori*-positive patients [90,91]. However, a Th1 to Th2 shift has been observed in patients with gastric cancer and dysplasia [29]. In fact, a mixed Th1/Th2 immune response is associated with non-atrophic chronic gastritis, while a prevalent Th2 immune response, mostly IL-13-mediated, characterizes patients with intestinal metaplasia and intestinal-type gastric cancer [38]. While a Th1-mediated immune response is considered mostly proinflammatory, Th2 responses can be immunoregulatory and protective and have the ability to promote antibody-mediated immunity against extracellular parasites and bacterial infection [92,93]. In fact, the overexpression of IL-1β in the gastric epithelium can promote gastric inflammation, atrophy, and metaplasia [94], and the knocking out of IL-1 has been shown to be protective against pathologic processes induced by *H. pylori* PMSS1 [76]. Similarly, the transgenic expression of interferon (IFN)γ, stromal-derived factor (SDF)-1, or IL-8 (the latter in transgenic mice bearing a human IL-8 bacterial artificial chromosome), accelerates gastritis and metaplasia during *Helicobacter* infection [95,96,97]. Conversely, the administration of IL-33 triggers a Th2 immune response, promoting metaplasia in the stomach. In fact, IL-33 is required to induce metaplasia after acute parietal cell loss during the development of SPEM by promoting a Th2 inflammatory response, mainly mediated by IL-13, and the recruitment of eosinophils [25] and macrophages M2 [69,98].

A seminal study in 2018 revealed that Th2, and notably not Th1 or Th17, immune responses are responsible for mediating the link between gastritis and gastric cancer; specifically, CTLA4 deficiency induces Th2-dependent immune dysregulation that results in the development of gastric cancer [99]. More recently, Noto et al. identified six cell subtypes (i.e., mast cells, CD19^+^ B cells, macrophages, group 2 innate lymphoid cells (ILC2s), as well as CD4^+^ and CD8+ T cells) as the main source of IL-13 in the context of gastritis, with mast cells being the greatest producers. In this study, the IL-13 receptor, an IL4Rα-containing heterodimer, is expressed on gastric epithelial cells, and IL-13 is crucial for the expansion of neck cells and subsequent SPEM by directly acting on epithelial cells. Indeed, IL4Rα-deficient mice develop gastritis, with immune cell infiltration and protein production resembling that found in wild-type controls but with no progression to SPEM, revealing the importance of IL-13 signaling during gastric carcinogenesis [98]. In line with these findings, a recent study identifying AQP5 in SPEM glands and at the base of incomplete intestinal metaplasia glands demonstrates that AQP5 expression is under the control of IL-13, further reinforcing the concept that this cytokine plays a fundamental role in the development of metaplasia and possibly, its progression to cancer [64]. Another recent study identified TNF^+^ Tregs as a source of IL-13 and proposes that IL-13 promotes self-renewal, migration and colony formation of gastric cancer cells via the phosphorylation of STAT3 [100].

### 3.2. IL-33 Is a Crucial Inducer of Th2 Responses Primarily, but Not Exclusively, by Inducing IL-13

IL-33 is widely distributed throughout various organ systems, both in non-hematopoietic and hematopoietic cells, the latter particularly in restricted populations of professional antigen-presenting cells, such as macrophages [101,102]. IL-33 was initially associated with Th2 immunity, based on the expression of its cell-bound receptor, ST2L, on polarized Th2 lymphocytes [101] and more recently, on ILC2s [103], and its ability to effectively induce M1, but more commonly M2, macrophage differentiation [104]. Furthermore, IL-33 is also considered a master regulator for eosinophil activation and recruitment into the mucosa of gastrointestinal and respiratory tracts interacting with the external environment [101,105,106]. High serum IL-33 concentration is reported to be associated with a poor prognosis in gastric cancer patients [107], and in vitro experiments suggest that IL-33 confers chemo-resistance [108] and increases invasiveness [109] to gastric cancer cells.

While the importance of IL-13 in gastric metaplasia seems to be mostly related to mucus hypersecretion [110,111], which is a specific feature of SPEM [87,112], IL-33-dependent induction of IL-13 is required to promote metaplasia following parietal cell loss and chief cell transdifferentiation into a mucus-producing cell type. It has been recently observed that the loss of *GRIM-19*, a mitochondrial gene commonly downregulated during gastric carcinogenesis, promotes SPEM in an IL-33-dependent manner; mechanistically, GRIM-19 deficiency, through the upregulation of NK-kB, leads to the activation of the NLRP3 inflammasome, which in turn, induces IL-33 expression and release [113]. Interestingly, IL-33 receptor-deficient mice do not develop SPEM following L635 treatment; however, the administration of recombinant IL-13 to these mice restores SPEM development, suggesting a major role of IL-13 in the pathogenesis of SPEM and for IL-33 as its upstream inducer [69]. In addition, IL-33-driven M2 macrophage polarization in L635-treated mice is associated with progression to a more advanced metaplasia, although not required for metaplasia induction [68,69]. Recently, epithelial-derived WAP four-disulfide core domain 2 WFDC2 was shown to induce SPEM via an IL-33-dependent promotion of M2 macrophage polarization; specifically, using chemically induced gastritis mouse models, the absence of *Wfdc2* results in reduced M2 infiltrates and no progression to SPEM and dysplasia, while the administration of rWFDC2 or recombinant IL-33 restores the development of SPEM [114].

Furthermore, metaplasia is thought to serve a regenerative function in response to tissue damage and appears to facilitate epithelial repair [112]. In line with this concept, the IL-33/ST2 axis plays a critical role in gut mucosal wound healing by promoting epithelial repair and restitution, overall restoration of barrier integrity, and the resolution of inflammation. IL-33 can initially sustain inflammation in the colon of mice immediately after acute dextran sodium sulfate (DSS) challenge; however, its primary role is to promote mucosal wound healing during recovery [115]. As such, while the IL-33/IL-13 axis may be important in promoting pro-tumorigenic processes, it can also conceivably be critical for gastric epithelial repair. These findings are consistent with previous studies that associate decreased IL-33 in TFF2-deficient mice with defective metaplasia development and delayed downstream gastric epithelial repair [112,116,117]. Moreover, mice deficient for IL-33, ST2 and IL-13, after acute treatment with L635, show diffuse parietal cell loss and reduction in the zymogen granule maturation transcription factor *Mist1*, but chief cells fail to undergo complete differentiation [69], indicating the need for an appropriate cytokine response to promote transdifferentiation as part of the repair processes for the gastric epithelium. In fact, IL-33 has been proposed to function as an autocrine stimulus for gastric carcinogenesis. IL-33 and its receptor ST2 are found to co-localize in poorly differentiated human gastric cells; functionally, IL-33 self-stimulation promotes the survival and proliferation of cancer stem cells, supposedly in cooperation with the Wnt-axis, and confers resistance to chemotherapy [118].

### 3.3. Cell Types Associated with the Cascade of Events Leading to Gastritis, and Subsequently to Gastric Metaplasia

IL-33, in fact, has emerged as an essential mediator for the development of eosinophil-mediated allergic inflammation and asthma [105,106] and plays a pivotal role in eosinophil recruitment and helminth expulsion after parasitic hook worm infection [106]. The effects of IL-33 on the gastric immune compartment include massive infiltration of both eosinophils and M2 macrophages [69], further perpetuating a chronic inflammatory state. Importantly, the influx of eosinophils during gastric metaplasia and cancer development is thought to have both pathogenic as well as protective functions. A prior study investigating early gastric cancers showed the presence of tumor stromal eosinophils with morphologic evidence of activation, as well as the presence of tumor cells in intimate contact with activated eosinophils that results in focal cytopathic changes [119]. Eosinophil development and recruitment depend on IL-5, which represents the primary factor for eosinophil maturation and differentiation but is also important for their activation and recruitment. Chemokine ligand (CCL) 11 and CCL24, also known as eotaxin-1 and eotaxin-2, respectively, are eosinophil-specific chemokines that bind to the chemokine receptor, CCR3, expressed on the surface of eosinophils, and are critical for eosinophil recruitment [120,121]. Eosinophils are associated with chronic intestinal inflammation, and IL-33 induces eosinophil infiltration into the gut and a potent mucosal Th2 immune response [122]. In the stomach, eosinophils are present in inflammatory infiltrates within the gastric mucosa during *Helicobacter* infection [123,124]. Moreover, IL-33 activation and recruitment of eosinophils into the gastric mucosa during chronic inflammation is central for initiating the cascade of events promoting SPEM [25]. Eosinophils play an important role during the early events leading to gastritis/metaplasia; however, there are other cell types belonging to the innate immune system that are responsive to IL-33 and may be involved in the gastritis/metaplasia process, such as IL-33-activated M2 macrophages, that in turn are potent producers of IL-33. In fact, it is well established that IL-33 has the ability to increase overall macrophage numbers in the stomach and to polarize them to an alternatively activated M2 phenotype, albeit in the periphery (peritoneum) [117]. Mice lacking IL-33 or ST2 with advanced SPEM have markedly decreased numbers of M2 macrophages, but administering rIL-13, which acts downstream of the IL-33/ST2 axis, can revert this condition in ST2-deficient mice [69]. In the SAMP model of chronic gastritis, eosinophils appear to be the main cell type driving M2 macrophage activation, and depleting eosinophils has a similar effect as blocking IL-33 in regard to reducing gastric M2 macrophage activity, which in turn, can also release IL-33, resulting in a vicious cycle that fuels itself. [25]. On the other hand, eosinophils do not seem to be relevant in the L635-induced model of SPEM, which, differently from the spontaneous chronic model of inflammation, develops SPEM via chemically induced parietal cell obliteration. Such differences in the model used can also account for the different cell types mainly involved in the initiation and perpetuation of advanced SPEM [69].

It has been recently shown that IL-33-activated mast cells, together with tumor-associated macrophages, allow for the progression of tumor angiogenesis and are correlated with poor survival in gastric cancer patients [125]. Mast cells and eosinophils interact in a complex self-perpetuating cycle, wherein eosinophils produce mediators stimulating mast cell differentiation, activation, proliferation and survival [126]. In addition, activated mast cells release IL-5 and granulocyte–monocyte colony-stimulating factor that induce eosinophil recruitment and activation [127]. Furthermore, IL-33 stimulation, in vitro, induces mast cells to release IL-2, which, in turn, promotes the differentiation and expansion of ICOS^+^ Tregs that inhibit CD8^+^ T cells. Notably, in a xenograft gastric cancer model adopting NOD/SCID mice, this IL-33/IL-2-dependent activation of ICOS^+^ Tregs is shown to promote tumor growth and progression [128].

Finally, a subtype of the ILC family, ILC2s, are regulated by the transcription factor GATA3 [129] and are stimulated by IL-33, IL-25, and TSLP to provide an innate source of type 2 cytokines [130,131]. ILC2s are involved in tissue remodeling, mucus metaplasia, eosinophilia, and alternative macrophage activation through the production of type 2 cytokines [132]. The role of ILC2s in promoting SPEM is controversial. ILC2s are reported to be indispensable for the induction of SPEM following injury-mediated loss of gastric parietal cells, where they accumulate in an IL-33-dependent fashion. Specifically, ILC2s-dependent tissue repair processes of the gastric mucosa after injury entail a series of changes in both mesenchymal and hematopoietic cell compartments, which go through chief cell reprogramming, tuft cell and mucin-secreting foveolar cell proliferation, as well as immune cell recruitment [133]. In this same study, gastric ILC2s exhibit a specific, metaplasia-associated transcriptomic profile associated with the development of SPEM [133]. Conversely, in chronic gastritis-prone SAMP mice, the increase in ILC2 frequency is quite modest, and the precise contribution of ILC2s to the SAMP gastric phenotype remains unknown. In fact, SAMP mice lacking T/B cells but with intact ILC function (i.e., SAMP x *Rag2^−/−^* strain) have virtually normal stomachs, suggesting that the role of ILCs is negligible for SPEM development, perhaps because of their inability to mount adaptive immune responses and sustain a chronic inflammatory state [25].

**Figure 2:**
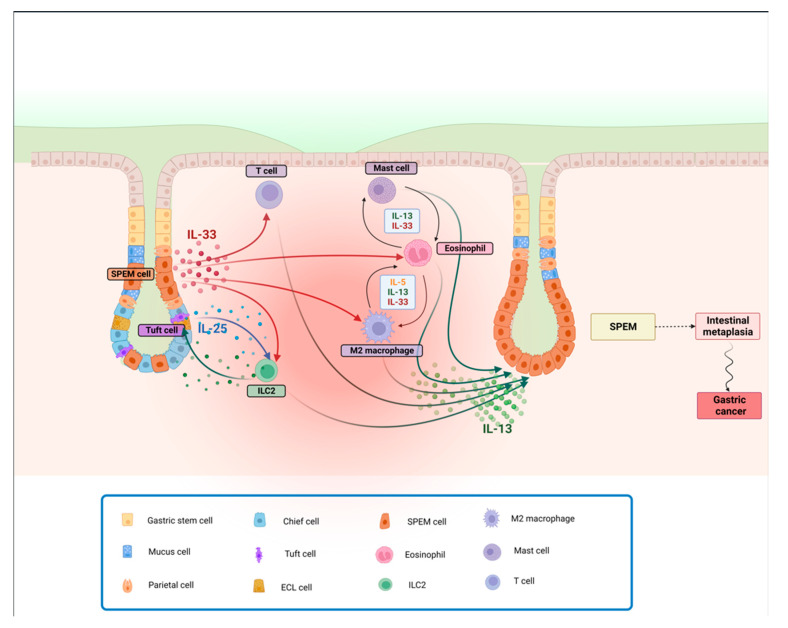
**Th2-depenent gastric metaplasia onset and progression to SPEM.** Following parietal cell loss, IL-33 is released, promoting activation and recruitment of different cell types involved in Th2 immune responses. IL-25, released by Tuft cells, also contributes, along with IL-33, to potentially induce the activation and proliferation of ILC2s. M2 macrophages and eosinophils, in turn, produce IL-13 and more IL-33, self-sustaining a feed-forward cycle, stimulating mast cell activity. The resulting downstream release of IL-13 promotes the progression to SPEM, that, in the presence of a chronic inflammatory insult, can lead to intestinal metaplasia and cancer. Abbreviations: IL, interleukin; SPEM, spasmolytic polypeptide-expressing metaplasia.

Nonetheless, it has been reported that IL-33 induces IL-13 production from ILC2s to promote intestinal goblet cell differentiation within the colons of mice [134], indicating that the IL-33/IL-13 axis might play a relevant role in inducing intestinal metaplasia in the context of chronic gastritis. Since IL-33 is an alarmin released by damaged epithelial cells and activated macrophages in the context of Th2 responses, it accumulates in the inflamed gastric mucosa, where it activates ILC2s to produce IL-13 [135]. In a recent paper, O’Keefe et al. showed that ILC2-derived IL-13 stimulates the expansion of tuft cells and induces them to produce IL-25, which in turn further promotes the activity of ILC2s. This feed-forward circuit is thought to facilitate both SPEM development and its subsequent transition to gastric cancer. Indeed, tumor-bearing gp130^−/−^ mice, when treated with pharmacological inhibitors of IL-13 or IL-25, are shown to bear smaller tumors containing fewer ILC2 and tuft cells. It is also suggested that, in patients with intestinal-like gastric cancer, a higher expression of gene signatures for ILC2s and tuft cells strongly correlates with reduced survival, further reinforcing the notion that this Th2 circuit contributes to the development and progression of gastric cancer, [136]. Furthermore, recent findings indicate a higher density of intratumoral IL-25-expressing macrophages that positively correlates with overall patient survival, possibly by regulating the T effector/T reg ratio [137] (Figure 2).

## 4. Conclusions

Increasing evidence supports Th2 immune responses and their associated cytokines to represent key players in the development of metaplasia [117] and may serve as important targets for the treatment and prevention of gastritis and gastric metaplasia. In fact, targeting cytokines, such as IL-33, IL-25, IL-13 and IL5, is effective in modulating the downstream recruitment and activation of immune cells, such as eosinophils, M2 macrophages, mast cells, and in some instances, ILC2s, therefore can be beneficial in preventing/reducing gastritis and downstream gastric metaplasia. Moreover, taking into account the impact of chronic inflammation in promoting dysregulated tissue repair processes resulting in metaplastic events, murine models, such as SAMP mice, that develop a spontaneously occurring phenotype and more closely resemble the human setting (without chemical, immunologic, or genetic manipulation), can prove to be very useful to thoroughly dissect the contribution of specific mediators and/or cell populations prior to, and during, the progression of disease. Other models that fully develop or can be induced to progress toward gastric dysplasia and/or advanced gastric cancer will also provide the appropriate tools to further understand the pathogenesis and treatment of this devastating condition.
